# Australian Hospitals

**Published:** 1906-02-24

**Authors:** 


### AUSTRALIAN HOSPITALS.

### VOLUNTARY SUPPORT LANGUISHING.

We have received an interesting letter from a correspondent, whose name we have not authority to give, but who is actively interested in the management of Australian hospitals, from which we extract the following:—

"I am grateful to you for your letter of December 1st, and for the courtesy that has always met any communication that I have had occasion to make concerning 'Burdett's Hospitals and Charities' which is highly valued here. . . . How you manage to worry out those relative costs passes my understanding. It may be easy when the lines are even, but here, where every institution follows its own wilful way of presenting its expenditure, it must be very difficult.

"The bursting of the financial boom of this country is affecting the hospitals greatly. Only in Melbourne, Sydney, and Brisbane are the large hospitals making any pretence of the voluntary system, and I fear that we will shortly fall on the rates and taxes, while (in my humble opinion) the other cities will presently follow, although there is much more money there than here.

"In Sydney the two large hospitals have been receiving £1 to the £1 subsidy up to £4,000, plus 3s. 6d. per day for destitute patients sent in by the Government officer, and these last year exceeded half of the total number.

"From July 1st this year there is a new departure at the Sydney Hospital. The Government continues to subsidise £1 to the £1 up to £4,000, and in addition they will allow £35 per bed up to 298 beds in lieu of the old destitute patients allowance of 3s. 6d. per day!

"That is fairly liberal you will admit, but this kind of prop must kill the little voluntary giving that remains."

